# Modulation of VEGF-induced migration and network formation by lymphatic endothelial cells: Roles of platelets and podoplanin

**DOI:** 10.1080/09537104.2017.1336210

**Published:** 2017-07-20

**Authors:** Stacey A. Langan, Leyre Navarro-Núñez, Steve P. Watson, Gerard B. Nash

**Affiliations:** Institute of Cardiovascular Sciences, College of Medical and Dental Sciences, University of Birmingham, Birmingham, UK

**Keywords:** CLEC-2, lymphangiogenesis, platelets, podoplanin, VEGF

## Abstract

Lymphatic endothelial cells (LEC) express the transmembrane receptor podoplanin whose only known endogenous ligand CLEC-2 is found on platelets. Both podoplanin and CLEC-2 are required for normal lymphangiogenesis as mice lacking either protein develop a blood-lymphatic mixing phenotype. We investigated the roles of podoplanin and its interaction with platelets in migration and tube formation by LEC. Addition of platelets or antibody-mediated crosslinking of podoplanin inhibited LEC migration induced by vascular endothelial growth factors (VEGF-A or VEGF-C), but did not modify basal migration or the response to basic fibroblast growth factor or epidermal growth factor. In addition, platelets and podoplanin crosslinking disrupted networks of LEC formed in co-culture with fibroblasts. Depletion of podoplanin in LEC using siRNA negated the pro-migratory effect of VEGF-A and VEGF-C. Inhibition of RhoA or Rho-kinase reduced LEC migration induced by VEGF-C, but had no further effect after crosslinking of podoplanin, suggesting that podoplanin is required for signaling downstream of VEGF-receptors but upstream of RhoA. Together, these data reveal for the first time that podoplanin is an intrinsic specific regulator of VEGF-mediated migration and network formation in LEC and identify crosslinking of podoplanin by platelets or antibodies as mechanisms to modulate this pathway.

## Introduction

During development of the lymphatics, endothelial cells of the cardinal vein commit to a lymphatic phenotype, expressing a number of markers including vascular endothelial growth factor receptor 3 (VEGFR3) [1–3]. These lymphatic endothelial cells (LEC) then migrate away from the cardinal vein in a VEGF-C dependent manner [4] to form the primordial thoracic duct, acquiring podoplanin expression during the process. Further sprouting from these nascent structures and subsequent remodeling creates the rest of the lymphatic network.

Podoplanin is the only known ligand for the platelet receptor CLEC-2, and previous studies have shown that both are necessary for the correct development of the lymphatic vasculature [5–7]. Mutant mice lacking either of these proteins displayed a phenotype in which blood was detected in lymphatic vessels. Similarly, mice lacking signaling proteins known to be downstream of CLEC-2, including Syk, SLP-76, and PLCγ2, displayed a blood-lymphatic mixing phenotype [8–11]. The platelet-specific role for CLEC-2 was confirmed by the creation and studies of lymphangiogenesis in *Clec1b*^fl/fl^PF4-Cre mice [12,13]. These studies also showed that platelets from mice expressing CLEC-2 inhibited the migration and formation of networks of LEC on matrigel, while platelets isolated from mice lacking expression had a significantly weaker inhibitory effect in both assays [12,13].

In comparison with CLEC-2 in platelets, signaling downstream of podoplanin in LEC has not been well described. The cytoplasmic domain of podoplanin consists of only nine amino acids, but contains a sequence of three basic amino acids that are necessary for interaction with the ezrin/radixin/moesin (ERM) protein family [14,15]. ERM proteins connect membrane-bound proteins to the actin cytoskeleton and are involved in signaling pathways that control cell migration and adhesion [16]. Overexpression of podoplanin in Madin-Darby canine kidney (MDCK) cells promoted migration and increased the amount of active RhoA, which was associated with an increase in phosphorylation of ERM proteins, leading the authors to conclude that podoplanin phosphorylates ERM proteins via RhoA and its effector protein, Rho kinase (ROCK) [15]. Conversely, it has been shown that siRNA-mediated knockdown of podoplanin in LEC reduced the ability of these cells to migrate across a wound or form networks on Matrigel [17,18]. Podoplanin knockdown was associated with a reduction in active RhoA and treating LEC with an inhibitor of RhoA was able to prevent network formation [17]. However, podoplanin knockdown had no effect on the amount of phosphorylated nor basal ERM proteins in LEC [17].

VEGF-C is a critical regulator of lymphangiogenesis whose absence results in a lack of lymphatic vessels and embryonic lethality [19–21]. A similar phenotype was observed in mice that had a mutation encoding a tyrosine kinase inactive VEGFR3 [22]. Conversely, overexpression of VEGF-C can increase lymphatic growth in adult tissues and is also thought to promote inflammation-induced lymphangiogenesis in the adult [23,24]. VEGF-C has multiple splice variants and its affinity for VEGFR3 increases with each processing step. Mature, fully processed VEGF-C is able to bind to VEGFR2 as well as VEGFR3 [25]. Soluble VEGFR3 or VEGFR3-blocking antibodies prevent the maturation of lymphatic capillaries during embryonic development and the early postnatal period, but have no effect on established vessels [26,27]. However, administration of VEGFR2- or VEGFR3-blocking antibodies to adult mice prevents lymphangiogenesis in areas of wound healing [28]. LEC express both VEGFR2 and VEGFR3 [29] and interaction between VEGF-C and VEGFR3 [30], or between VEGFA and VEGFR2 [31] have both been reported to increase LEC migration.

The above suggests that podoplanin and VEGF-mediated signaling both regulate LEC migration and that both are required for mature lymphangiogenesis as well as embryonic formation of the lymphatics. However, the link between the two pathways has not been fully explored. Moreover, the role of platelets in formation of lymphatics and their ability to alter behavior of LEC suggests that crosslinking of podoplanin by CLEC-2 further regulates this axis.

To investigate further, we analyzed the effects of platelets, podoplanin crosslinking by antibodies, and podoplanin knockdown by siRNA on migration of LEC through micropore filters, and on the stable tube-like networks of LEC formed when they were co-cultured with human dermal fibroblasts. We found that antibody-mediated crosslinking of podoplanin inhibited migration and network formation by LEC in a similar manner to platelets. Reduction in expression of podoplanin independently reduced the effects of platelets and of responses to VEGFA or VEGFC on migration, but not basal migration or increases induced by other growth factors. Inhibition of RhoA or Rho-kinase also inhibited VEGF-induced migration, but crosslinking of podoplanin had no additional effects, suggesting that podoplanin was linked to RhoA downstream of VEGFR signaling. Thus, podoplanin intrinsically and specifically regulates responses to different VEGF family members, while crosslinking of podoplanin through platelets or otherwise likewise may influence the development and stability of forming lymphatic vessels.

## Materials and methods

### Ethics statement

The work described here was performed with the ethical approval of the Science, Technology, Engineering, and Mathematical Ethical Review Committee of the University of Birmingham. All blood donors were adult volunteers who had given informed consent. All animal work was performed under a UK Home Office license.

### Cell culture

Human dermal primary LEC and human primary dermal fibroblasts (HDF) were from PromoCell (Heidelberg, Germany). The HMEC-1 cell line was obtained from the Centre for Disease Control and Prevention (CDC; Atlanta, Georgia, USA). LEC and HDF were cultured in MV2 and FGM2, respectively (both PromoCell). HMEC-1 were cultured in M199 (Gibco, Paisley, UK) that was supplemented with 20% foetal calf serum (Sigma, Poole, UK). With the exception of siRNA transfections, cells were treated with 2.5µg/ml amphotericin (Gibco), 100U/ml penicillin and 100µg/ml streptomycin (both Sigma). All cells were cultured at 37°C and 5% CO_2_ and 95% air.

### Platelet preparation

Whole blood was drawn into anticoagulant citrate phosphate dextrose adenine (CPDA; pH 7.4; Sigma) at a ratio of 1:9. Washed human platelets were isolated using theophylline, as previously described [32]. Mouse blood was drawn from the vena cava into 10% ACD. Washed platelets were isolated by centrifugation, and platelet activation was prevented using prostacyclin. The full methods have been described previously [33,34]. In some experiments, platelets were derived from mice deficient in CLEC-2 (*Clec1b*-/-) previously described [12]. Conditional deletion of CLEC-2 was achieved by insertion of loxP sites flanking exons 3 and 4 of the *Clec1b* gene (*Clec1b*fl/fl), using standard methods. Cre-mediated recombination of the *Clec1b*fl allele results in deletion of exons 3 and 4, and a frameshift in exons 5 and 6 *(Clec1b*fl/flPF4-Cre).

### siRNA transfections and flow cytometry

LEC were seeded onto 24-well plates in antibiotic-free medium. Transfections were performed the following day, when cells were around 80% confluent. Two validated siRNA duplexes targeting podoplanin (siRNA ID: 1. SASI_Hs01_00094891 and 2. SASI_Hs01_00192618) and a nonspecific control (Universal negative control #1SIC001) were purchased from Sigma. Duplexes were diluted in Optimem (Gibco) to give a final concentration of 10-70nM when added to cells. In a separate tube, 10% RNAiMAX lipofectamine (Invitrogen, Paisley, UK) in Optimem was prepared. Both solutions were incubated at room temperature for 10 minutes, then combined and incubated for a further 10 minutes. About 500µl diluted duplex was added to each well. The plate was incubated at 37°C and 5% CO_2_ for 4 hours, before the transfection mix was replaced with antibiotic-free medium and incubated for a further 24-72 hours.

Expression of podoplanin, VEGFR2, and VEGFR3 was quantified by flow cytometry. Cells were detached with Accutase (Gibco) and stained with PE-conjugated antibodies against human podoplanin (0.125µg; clone NZ-1.3; eBioscience, Hatfield, UK), VEGFR2 (1:7.5; 89106; R&D Systems, Abingdon, UK) or VEGFR3 (1:7.5; 54733; R&D Systems) or an appropriate control (0.125µg rat IgG_2a_; eBioscience; 1:7.5 mouse IgG_1_; Immunotools, Friesoythe, Germany). Samples were protected from light and incubated on ice for 40 minutes. Samples were diluted with 300µl PBS before analysis on a FACSCalibur flow cytometer (BD Bioscience, Oxford, UK). Data were analyzed using Summit v4.3 (Dako, Colorado, USA), to obtain values for percent positive (above isotype control) and median fluorescence intensity (MFI).

### Analysis of cell migration

3x10^4^ endothelial cells were seeded onto polyethylene terephthalate cell culture inserts with 8µm pores (BD Biosciences). These were placed in 24-well plates with culture medium, 300ng/ml VEGF-C, 30ng/ml VEGF-A (both R&D Systems), 10ng/ml basic fibroblast growth factor (bFGF; Peprotech; London, UK), or 20ng/ml epidermal growth factor (EGF; Sigma) in the lower chamber of the well. The plates were incubated for an hour at 37°C and 5% CO_2_ before the addition of washed platelets. In podoplanin-crosslinking experiments, LEC were incubated on the inserts for 30 minutes at room temperature before addition of 2µg/ml rat IgG2a or anti-human podoplanin (NZ-1.3; eBioscience, Hatfield, UK). The plate was incubated at room temperature for 30 minutes before addition of 30µg/ml anti-rat IgG_2a_ (eBioscience) to induce crosslinking.

In experiments where the effects of antibodies against VEGF-receptors were tested, endothelial cells were seeded for 30min and antibodies against VEGFR2 (IMC-1121b), VEGFR3 (IMC-3C5; both 5µg/ml, gifts of Eli Lilly and Company, New York, USA), or both were added for 30min before addition of VEGFA or VEGFC.

Where inhibitors of RhoA or ROCK were used, podoplanin was crosslinked when appropriate and the plate was incubated at room temperature for 30 minutes before the addition of 4µg/ml CT04 (Cytoskeleton Inc., Colorado, USA) or 100µM Y27632 (Calbiochem, Nottingham, UK), respectively, and incubated for 12 or 24 hours. Initial studies compared effects of 10 and 100µM Y27632 directly on LEC migration and showed a dose-dependent effect which was greater and significant at the higher concentration; and this concentration was used thereafter and in experiments reported below.

At the end of all migration experiments, cells were washed with PBS, fixed with 2% formaldehyde and their nuclei stained with 2µg/ml bisbenzimide (Sigma). The stained nuclei were imaged using an invert fluorescent microscope (AxioVert 200M; Zeiss, Hertfordshire, UK). The numbers of cells above and below the insert were counted in 12 fields per filter, with each condition in each experiment tested in duplicate wells and averaged. Percentage transmigration was calculated as follows:

$$Percentage\;migration=\;\frac{Number\;\;of\;\;migrated\;\;cells}{Total\;\;number\;\;of\;\;cells}\times 100$$

### Formulation of tubes in LEC–HDF co-cultures

3x10^4^ HDF were seeded onto wells of a 12-well plate and cultured until confluent (typically four days). LEC were stained with 5µM Cell Tracker Green (Life Technologies, Paisley, UK). 3x10^4^ stained LEC were seeded onto each confluent well of HDF. The co-cultures were maintained in MV2 medium with or without 300ng/ml VEGF-C for three days. Images were taken using the 10x objective of an invert fluorescent microscope (Olympus, Southend-on-Sea, UK). 2x10^8^ washed platelets were added to the co-cultures, or podoplanin was crosslinked, as described above. The cells were co-cultured for a further 24 hours before additional fluorescent images were taken. Images were analyzed offline using ImageJ 1.49 (NIH, Bethesda, Maryland, USA). The segmented line tool was used to draw along each tube; the length of the tube was then measured.

### Statistical analysis

Variation between multiple treatments was evaluated using analysis of variance (ANOVA). In cases showing significant variation, post hoc comparisons were made to control by Dunnett test or between conditions by Bonferroni test. Effects of single treatments were analyzed by paired t-test compared to controls.

## Results

### Characterization of podoplanin expression by lymphatic endothelial cells

**Figure 1.**
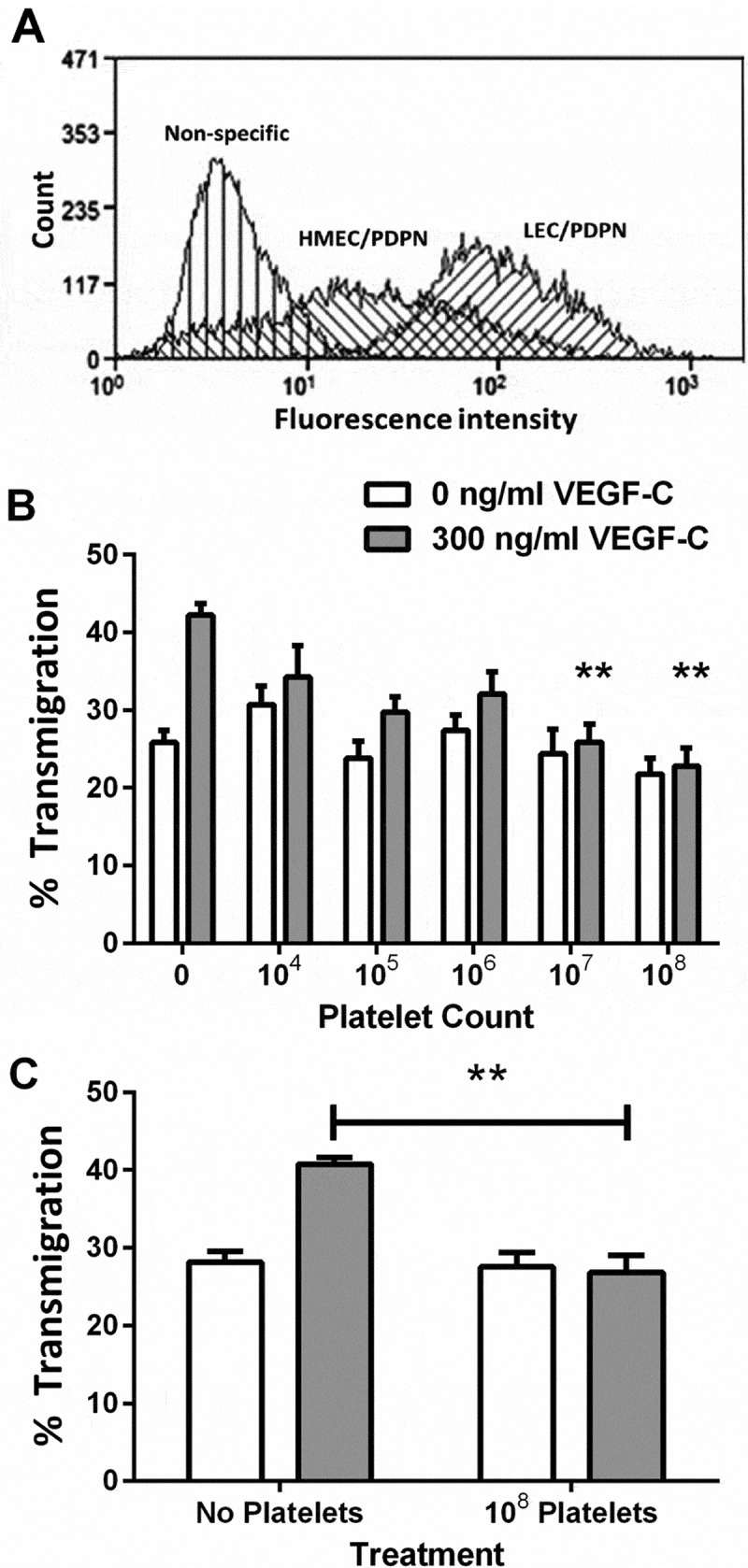
Expression of podoplanin on lymphatic endothelial cells and effect of platelets on their migration. A) Flow cytometer, frequency distributions of fluorescence intensity for LEC and HMEC-1 labeled with antibody against podoplanin (PDPN). B) Effect on migration when different numbers of platelets were added to LEC which had been settled onto a Transwell filter, with or without treatment with VEGF-C (300 ng/ml). The percentage of the LEC which had transmigrated after 24h was analyzed. Data are mean ± SEM from four experiments. ANOVA showed significant effects of VEGF-C and of platelet count (*p* < 0.01 in each case). **=*p* < 0.01 compared to 0 platelets by Dunnett’s test, for VEGF-C-treated LEC. C) Effect on migration when platelets (10^8^) were added to HMEC-1 which had been settled onto a Transwell filter, with or without treatment with VEGF-C (300 ng/ml). The percentage of the HMEC-1 which had transmigrated after 24 h was analyzed. Data are mean ± SEM from three experiments. ANOVA showed significant effects of VEGF-C and of platelets (*p* < 0.01 in each case). **=*p* < 0.01 compared to 0 platelets by Dunnett’s test, for VEGF-C-treated LEC.

Flow cytometry showed that LEC were >90% positive for expression of podoplanin and HMEC-1 ~50% positive, although a smaller proportion actually fell under the nonspecific labeling peak (Figure 1A). The effects of siRNA transfection at 30-70nM on podoplanin expression by LEC were similar, as were the effects of the two different siRNAs. After 24h, we found approximately 50% reduction in surface expression and this remained essentially constant up to 72h. Combining the two siRNA did not reduce expression further. For subsequent functional experiments, we used siRNA1 at 30nM and carried out functional analyses 48h after transfection when MFI was 43 ±4% of control (mean ± SEM, n=6).

### Effect of platelets on migration of lymphatic endothelial cells

The effect of platelets on LEC migration was assessed using the transfilter migration assay. Washed human platelets had no effect on migration in the absence of VEGF-C but inhibited the increase in migration induced by VEGF-C in a count-dependent manner (Figure 1B). Platelets also inhibited VEGF-C-promoted migration of HMEC-1 (Figure 1C), an immortalized endothelial cell line which we and others [35] have found to express podoplanin. In each type of cell, 10^8^ platelets completely inhibited the pro-migratory effect of added VEGF-C. However, the effect of VEGF was itself greater for LEC (increase above baseline 64 ± 4%; mean ± SEM of four independent experiments) than for HMEC-1 (increase above baseline 46 ± 8%; mean ± SEM of three independent experiments). This suggests that the effect of VEGF-C was greater for cells with greater expression of podoplanin. The more puzzling observation that platelets were able to completely down-regulate VEGF-mediated migration even for HMEC-1 that expressed podoplanin at a lower and non-uniform level would seem to require that only cells expressing podoplanin above a threshold level could respond to VEGF-C. We thus examined the role of podoplanin in supporting the effect of VEGF-C further.

### Effects of crosslinking or knock-down of podoplanin on migration of LEC induced by VEGF

**Figure 2.**
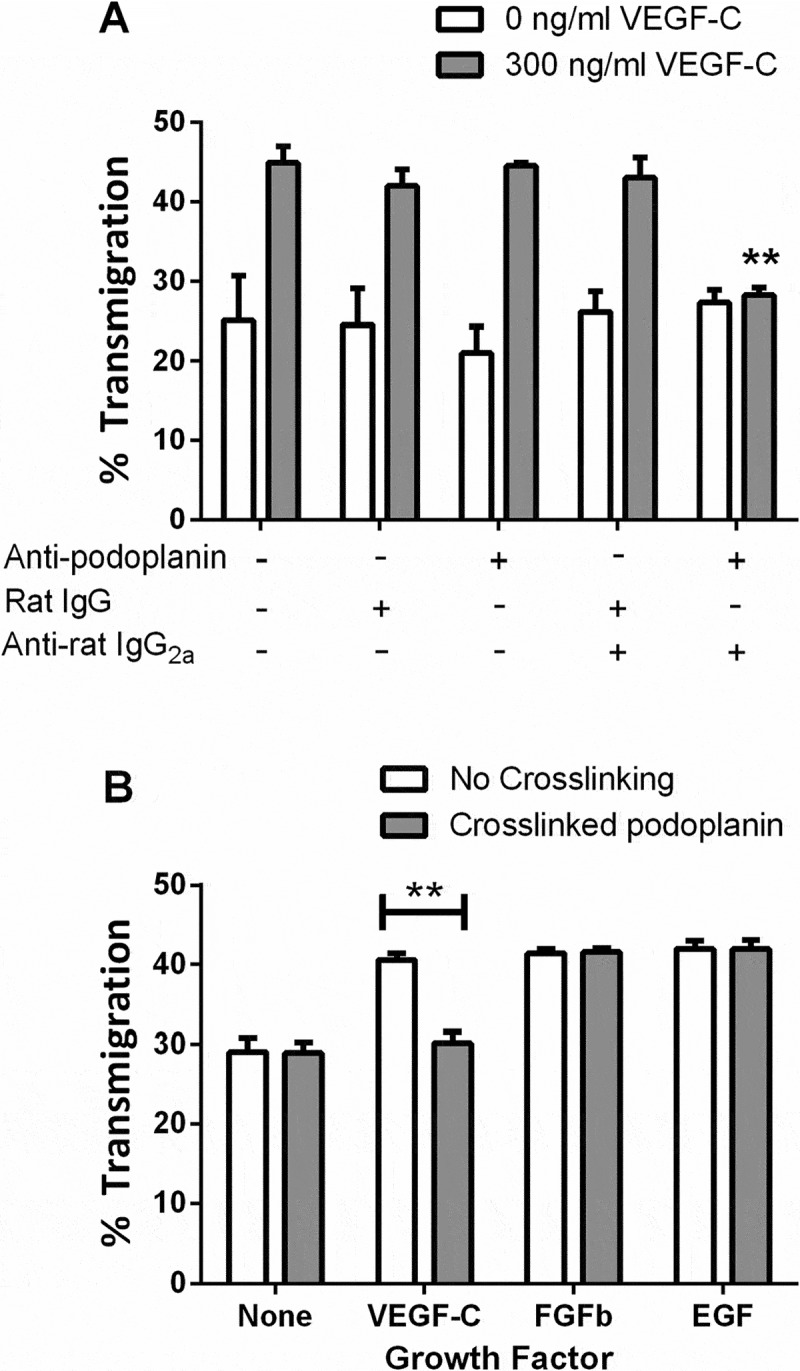
Effects of podoplanin crosslinking on LEC migration. A) LEC settled onto a Transwell filter were treated with antibody against podoplanin or a nonspecific IgG, with or without a crosslinking secondary antibody, with or without treatment with VEGF-C (300 ng/ml). The percentage of the LEC which had transmigrated after 24 h was analyzed. Data are mean ± SEM from three experiments. ANOVA showed significant effects of VEGF-C and of antibody treatment (*p* < 0.01 in each case). **=*p* < 0.01 compared to no antibodies by Dunnett’s test, for VEGF-C-treated LEC. B) LEC settled onto a Transwell filter were treated with VEGF-C (300 ng/ml), FGFb (10 ng/ml) or EGF (20 ng/ml), with or without antibodies to induce crosslinking of podoplanin. The percentage of the LEC which had transmigrated after 24h was analyzed. Data are mean ± SEM from at least three experiments. **=*p* < 0.01 by paired t test.

Platelets may have exerted their effects on LEC through clustering podoplanin. We thus tested the effect of crosslinking podoplanin, using an antibody against podoplanin (clone NZ-1.3) and an appropriate secondary antibody. Podoplanin crosslinking inhibited LEC migration in the presence of VEGF-C (Figure 2A). Consistent with the effects of platelets, crosslinking podoplanin had no effect on LEC migration in the absence of VEGF-C. Anti-podoplanin or nonspecific rat IgG alone had no effect on LEC migration; similarly, combining nonspecific IgG with the secondary antibody did not alter migration (Figure 2A).

To confirm whether the effect of podoplanin crosslinking was specific to effects of VEGF on migration, transfilter assays were performed in the presence of VEGF-C, bFGF, and EGF. All three growth factors were able to promote LEC migration, but podoplanin crosslinking only inhibited migration in the presence of VEGF-C (Figure 2B).

**Figure 3.**
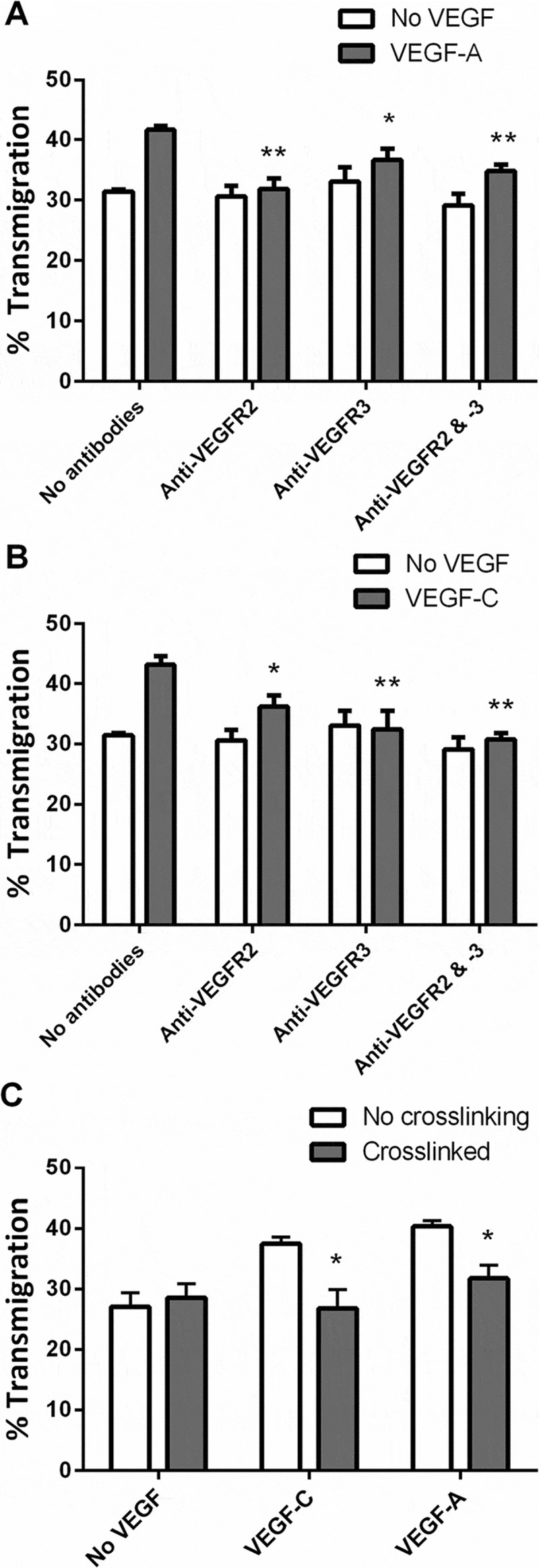
Effects of different types of VEGF on LEC migration: inhibition by antibodies against VEGF-R and by podoplanin crosslinking. A,B) Antibodies against VEGFR-2, VEGFR-3, or both were added to LEC settled onto a Transwell filter, with or without treatment with (A) VEGF-A (30 ng/ml) or (B) VEGF-C (300 ng/ml). The percentage of the LEC which had transmigrated after 24h was analyzed. Data are mean ± SEM from four experiments. ANOVA showed significant effects of VEGF and of antibody treatment (*p* < 0.01 in each case). *=*p* < 0.05, **=*p* < 0.01 compared to no antibodies by Dunnett’s test, for VEGF-treated LEC. C) LEC settled onto a Transwell filter were treated with VEGF-A or VEGF-C, with or without antibodies to induce crosslinking of podoplanin. The percentage of the LEC which had transmigrated after 24 h was analyzed. Data are mean ± SEM from six experiments. ANOVA showed significant effects of VEGF and of crosslinking (*p* < 0.01 in each case).). *=*p* < 0.05, **=*p* < 0.01 compared to no crosslinking by Bonferroni test, for VEGF-treated LEC.

Since these results implied that podoplanin crosslinking by platelet or antibodies inhibited VEGF-mediated signaling, we investigated the specificity of the effects for different members of the VEGF family and their receptors. VEGF-A and VEGF-C each promoted transmigration of LEC (Figure 3A,B). Antibody against VEGFR2 effectively inhibited the increase induced by VEGF-A and partially reduced the effect of VEGF-C, whereas antibody against VEGFR-3 effectively inhibited the increase induced by VEGFC and partially reduced the effect of VEGFA (Figure 3A, B). Combination of antibodies did not reduce migration further than the more effective antibody alone. Next, we tested the effects of crosslinking podoplanin for each isoform. Crosslinking reduced migration promoted by either VEGF-A or VEGF-C to the basal unstimulated level (Figure 3C). These results suggest that pro-migratory effects of VEGF-A and VEGF-C and signals through VEGFR-2 and VEGFR-3 were inhibited by crosslinking podoplanin.

**Figure 4.**
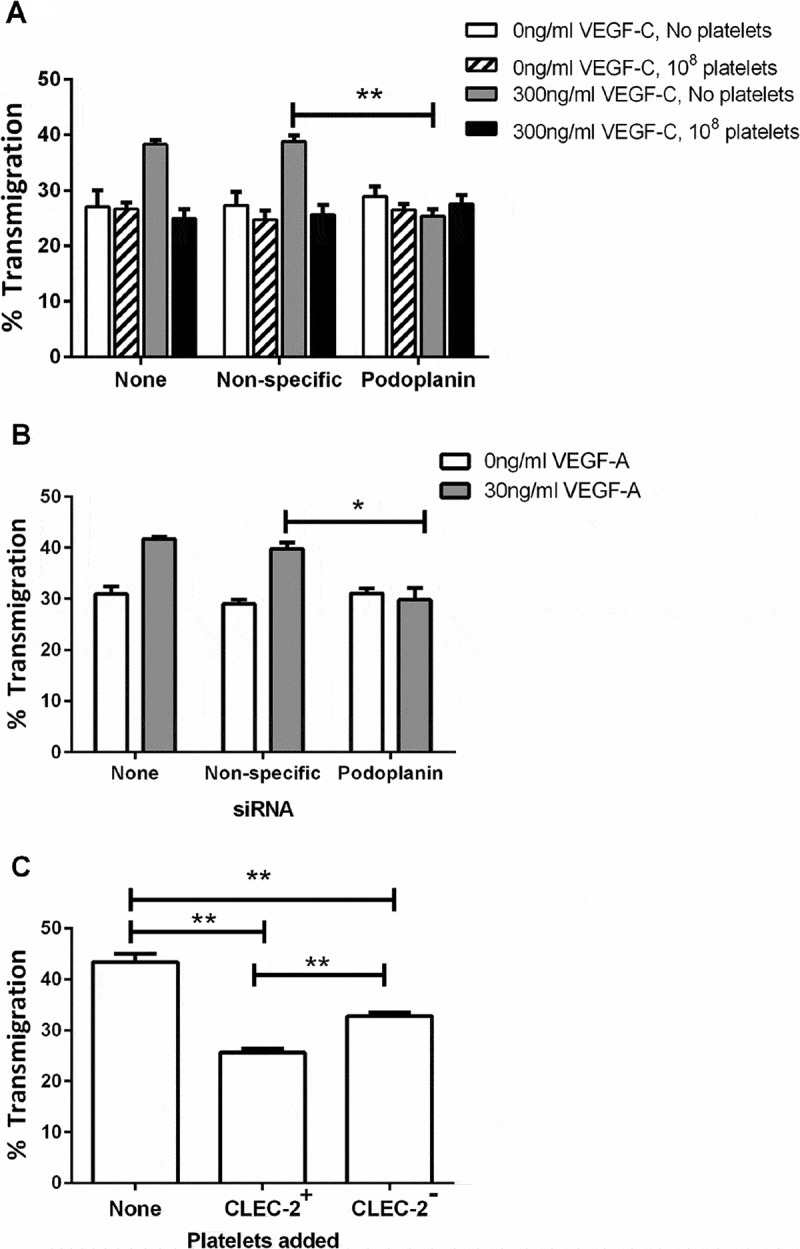
Effects of podoplanin knockdown or of platelets lacking CLEC-2 on LEC migration. A) LEC settled onto a Transwell filter were treated with or without platelets (10^8^), with or without siRNA targeting podoplanin, with or without treatment with VEGF-C (300 ng/ml). The percentage of the LEC which had transmigrated after 24 h was analyzed. Data are mean ± SEM from three experiments. ANOVA showed significant effects of VEGF-C, siRNA treatment and platelet treatment (*p* < 0.01 in each case). **=*p* < 0.01 compared to nonspecific siRNA by Dunnett’s test, for VEGF-C-treated LEC. B) LEC settled onto a Transwell filter were treated with or without siRNA targeting podoplanin, with or without treatment with VEGF-A (30 ng/ml). The percentage of the LEC which had transmigrated after 24 h was analyzed. Data are mean ± SEM from at least three experiments. ANOVA showed significant effects of VEGF-A and of siRNA treatment (*p* < 0.01 in each case). **=*p* < 0.01 compared to nonspecific siRNA by Dunnett’s test, for VEGF-A-treated LEC. C) Platelets (10^8^) were added to LEC which had been settled onto a Transwell filter in the presence of VEGF-C (300 ng/ml). The platelets were from *Clec1b*fl/fl (CLEC-2^+^) or *Clec1b*fl/flPF4-Cre (CLEC-2^−^) mice. Wells without platelets received 100μl Ca2+/Mg2+-free sterile PBS. The percentage of the LEC which had transmigrated after 24h was analyzed. Data are mean ± SEM from at least four experiments. ANOVA showed that platelets had a significant effect (*p* < 0.01). ** = *p* < 0.01 by Bonferroni post-test.

To further test the role for podoplanin in cell migration, siRNA was used to deplete podoplanin on LEC. The podoplanin-knockdown cells were then used in transfilter assays, with or without added platelets. In the absence of VEGF-C, neither podoplanin knockdown nor platelets had an effect on basal migration (Figure 4A). In the presence of VEGF-C, platelets inhibited migration of LEC that had not been transfected and cells that had been transfected with nonspecific siRNA. When podoplanin siRNA was transfected, VEGF-C no longer promoted that migration and platelets were not able to further inhibit migration (Figure 4A). Similarly, VEGF-A was not able to promote migration of LEC after podoplanin knockdown (Figure 4B). The effect of platelets was likely due to ligation of podoplanin by CLEC-2. We tested this possibility by comparing effects of platelets from mice expressing or lacking expression of CLEC-2 (*Clec1b*fl/fl or *Clec1b*fl/flPF4-Cre mice, respectively). We found that the murine CLEC-2^+^ platelets suppressed transmigration, and this effect was significantly less (but not abolished) with the platelets lacking CLEC-2 (Figure 4C). The results support the conclusions that effects of platelets through podoplanin were mediated through CLEC-2.

To determine whether the loss of effect of VEGF was due to a change in receptor expression, flow cytometry was used to quantify cell surface levels of VEGFR2 and VEGFR3. We found that transfection of siRNA into LEC had no significant effect on surface expression of VEGFR2 or VEGFR3 (values 85±7% or 84±13% of control; mean ±SEM, n=3). These results suggest that podoplanin is a novel regulator of VEGF-mediated responses in LEC.

### Effects of platelets and podoplanin crosslinking on stability of LEC networks

**Figure 5.**
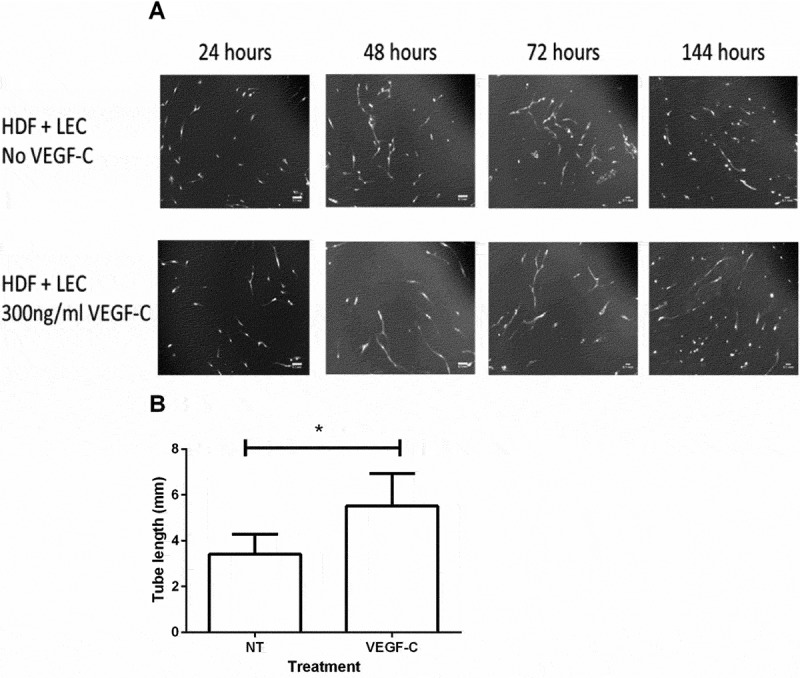
Time course of tube formation by LEC cultured with fibroblasts. A. Human dermal fibroblasts were grown to confluence on 12-well plates. 3 × 10^4^ LEC were stained with 5 µM Cell Tracker Green and seeded onto the HDF monolayer in the presence of culture medium with (lower panels) or without (upper panels) VEGF-C (300 ng/ml). Cultures were maintained at 37°C and 5% CO_2_ and imaged using a fluorescence microscope at 24 hour intervals. Images are representative of three independent experiments. Scale bars represent 100 µm. B. Quantitation of tube length after 72 h with or without VEGF-C. Data are mean ± SEM from three experiments. *p < 0.05 by paired t-test.

**Figure 6.**
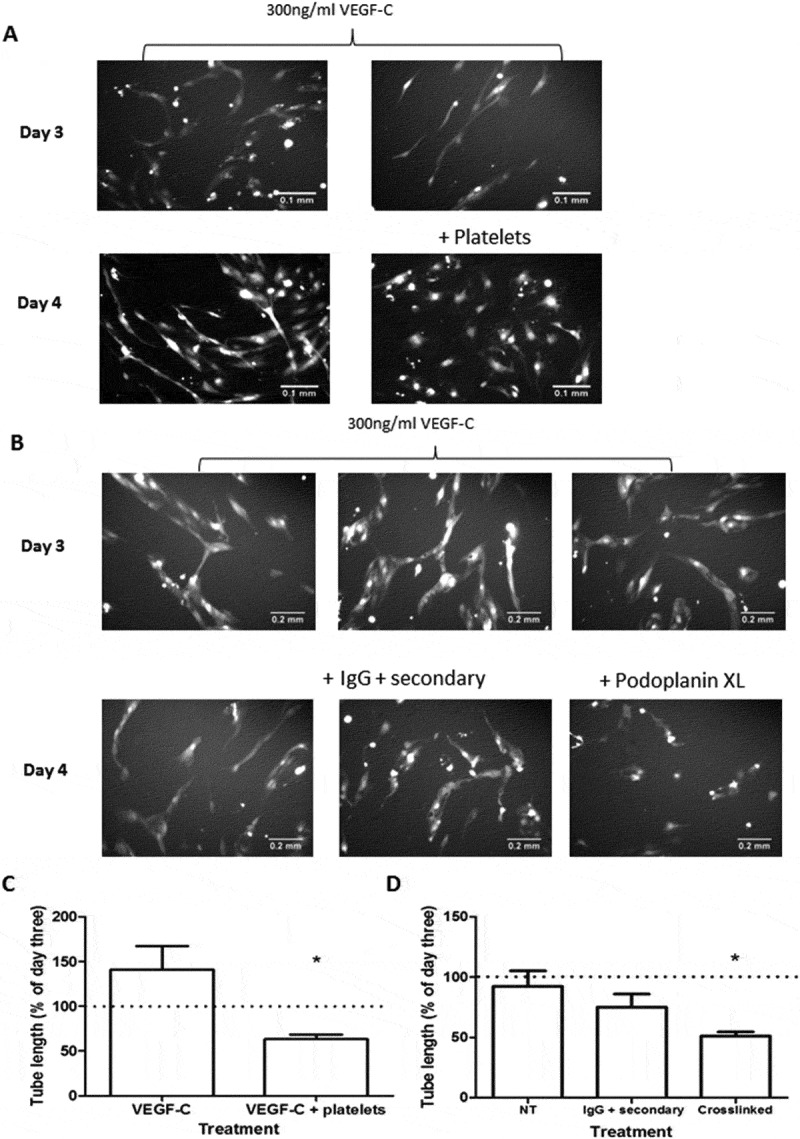
Effects of Platelets and podoplanin crosslinking on networks of LEC formed on fibroblasts. A) Networks of LEC formed after 3 or 4 days of co-culture with HDF plus VEGF-C (300 ng/ml), with or without addition of washed human platelets after 3 days. Images are representative of three independent experiments. B) Networks of LEC formed after 3 or 4 days of co-culture with HDF plus VEGF-C (300 ng/ml), with or without addition of antibodies that were nonspecific or which induced crosslinking of podoplanin. Images are representative of two or more independent experiments. C) Quantitation of tube length from section A at day 4 in experiments where platelets were added at day 3 to networks treated with VEGF-C. Data are mean ± SEM from three experiments, expressed relative to tube length at day 3. *p < 0.05 by paired t-test. D) Quantitation of tube length from section B at day 4 in experiments where antibodies that were nonspecific or which induced crosslinking of podoplanin were added at day 3 to networks treated with VEGF-C. Data are mean ± SEM from two to five experiments, expressed relative to tube length at day 3. *p < 0.05 by paired t-test compared to untreated.

Having shown that platelets and podoplanin crosslinking modulate VEGF-mediated LEC migration, we also examined their effects on networks of LEC, adapting a method described for vascular endothelial cells [36–38] but not yet reported for LECs. We found for the first time that stable networks of LEC could be established on monolayers of fibroblasts. Networks of LEC formed within 72 hours, with little further growth beyond this time (Figure 5A). With added VEGF-C, networks tended to be more stable and extensive than without (Figure 5B). It was shown previously that fibroblasts in this co-culture assay produced VEGF and that inhibition of VEGF-R reduced tube formation even when no exogenous VEGF was added [36]. This indicates that any effect of platelets or podoplanin on networks could not be studied independent of VEGF. Since VEGF is intrinsic to the assay, we added VEGF-C to obtain as standard conditions as possible under which to test the effects podoplanin ligation or crosslinking. To assess the effects of platelets on tube formation, LEC were co-cultured with HDF and VEGF-C for three days to allow networks to form and then incubated with platelets for 24 hours. The LEC became rounded and the previously detected networks disintegrated (Figure 6A). Similarly, podoplanin crosslinking induced network disintegration, which was not seen in wells treated with a rat IgG and appropriate secondary (Figure 6B). Quantitation of tube length in both conditions confirmed that platelets and podoplanin crosslinking significantly disrupted previously formed networks of LEC within 24 hours (Figure 6C, D). Thus, podoplanin appears able to regulate stability of lymphatic vessels as well as cell migration required for their formation.

### Investigation of signaling downstream of VEGF-R and podoplanin

**Figure 7.**
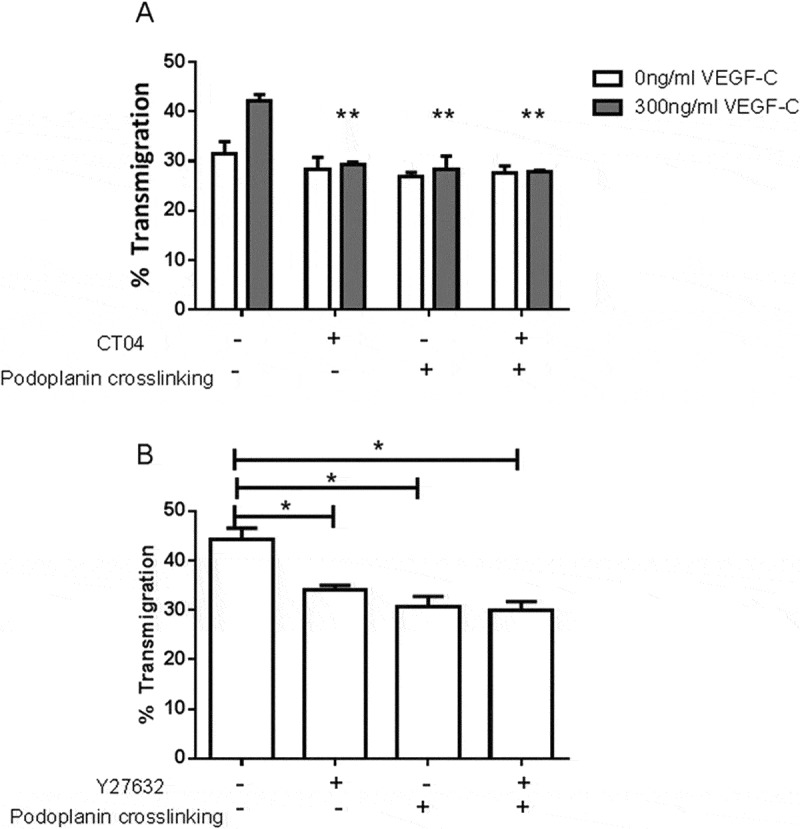
Effects of inhibitors of RhoA signaling combined with podoplanin crosslinking on LEC migration. A) LEC settled onto a Transwell filter were treated with or without RhoA inhibitor CT04 (4 µg/ml), with or without antibodies that induced podoplanin crosslinking, with or without VEGF-C (300 ng/ml). The percentage of the LEC which had transmigrated after 24 h was analyzed. Data are mean ± SEM from three experiments. ANOVA showed significant effects of VEGF-C (*p* < 0.05) and treatment (*p* < 0.01). **=*p* < 0.01 compared to no treatment by Dunnet test for VEGF-C-treated cells. B) LEC settled onto a Transwell filter were treated with or without ROCK inhibitor Y27632 (100 µM), with or without antibodies that induced podoplanin crosslinking, with VEGF-C (300ng/ml). The percentage of the LEC which had transmigrated after 24 h was analyzed. Data are mean ± SEM from three or more experiments. ANOVA showed significant effect of treatment (*p* < 0.01). **=*p* < 0.01 compared to no treatment by Dunnett´s test.

Since podoplanin appeared to modify signals downstream of VEGF-R, we investigated whether podoplanin was linked to, or independent of, small GTPase signaling, also likely to regulate migration responses. Inhibitors of RhoA (CT04) and of Rho kinase (Y27632) were used in combination with podoplanin crosslinking. We found that CT04 had little effect on unstimulated migration but inhibited migration induced by VEGF-C in a similar manner to podoplanin crosslinking (Figure 7A). However, CT04 did not further reduce migration when combined with podoplanin crosslinking. Similarly, Y27632 inhibited migration induced by VEGF-C, but did not further reduce migration when combined with podoplanin crosslinking (Figure 7B). These results suggested that podoplanin acted downstream of VEGF-C receptor(s) and upstream of RhoA in effecting the migratory response.

## Discussion

We have shown that addition of platelets to LEC or antibody-induced crosslinking of podoplanin on LEC both suppresses the pro-migratory effects of VEGF. On the other hand, reduction in expression of podoplanin induced an equivalent suppression of the response to VEGF. These treatments inhibited responses to both VEGF-A and VEGF-C, but none affected the substantial basal migration in the absence of VEGF, nor did crosslinking inhibit increases in migration induced by bFGF or EGF. Blockade of receptors VEGFR2 and VEGFR3 indicated that these receptors primarily supported the responses to VEGF-A and VEGF-C, respectively, although response to VEGF-C appeared to utilize both receptors. Thus, podoplanin is a regulator of LEC migration specific to VEGF and is able to modulate signaling through VEGFR-2 and VEGFR3.

Platelets were also able to suppress migration of HMEC-1, an immortalized endothelial cell line derived from dermal tissue which express podoplanin, albeit at a lower level than LEC. Interestingly, the pro-migratory effect of VEGF-C was less for these cells than LEC, but platelets returned migration of both to baseline level. Both cell types had significant basal migration without VEGF, and for LEC at least, this was not dependent on podoplanin expression or crosslinking. It seems, however, that the addition of VEGF increased transmigration by a degree depending on the level of podoplanin expressed or the proportion of cells expressing podoplanin. Subsequently, if the responding cells expressing adequate podoplanin became non-responders when podoplanin was ligated, the overall population migration would return to the level without VEGF-C for either LEC or HMEC-1. Put the other way, the trends suggest that only those cells expressing podoplanin above a certain level would respond to VEGF and have that response modified by ligation of podoplanin. These trends thus further support the key role of podoplanin in regulating the response to VEGF-C.

We developed a “lymphatic network” formation assay, based on the co-culture of LEC and HDF. This model was based on one previously used with vascular endothelial cells (VEC) in the study of angiogenesis [36–38], and we report the first description of formation of comparable stable networks for LEC. Addition of platelets to these networks or crosslinking of podoplanin caused their disruption. Thus, overall, crosslinking of podoplanin mimicked responses to platelets closely, and indeed, the effect of platelets on migration was lost after reduction in expression of podoplanin by siRNA. Migration of LEC was also suppressed when murine platelets were added, but the effect was significantly reduced when the platelets were derived from mice lacking expression of CLEC-2. Taken together, the results strongly suggest that the effects of platelets were mediated through crosslinking of podoplanin by CLEC-2, which has previously been shown to be able to fulfill that function [12,39].

Downstream of VEGFR, inhibition of RhoA, or of ROCK nullified migratory responses to VEGF-C. Interestingly, crosslinking of podoplanin had no further effect when combined with these inhibitors. Rho GTPases are well-described regulators of cell motility [40], and these results indicate that podoplanin regulated signaling through RhoA.

Our findings are broadly consistent with previous observations on behavior of LEC. We recently reported that platelets or crosslinking of podoplanin inhibited LEC migration in presence of VEGFC and short-term formation of networks on Matrigel [12]. Osada et al., found similar effects of platelets on these responses and showed that soluble agents released by platelets activated through GpVI ligation could also inhibit migration and network formation on Matrigel [13]. The relevance of the effects of substances released through GpVI ligation to the effects of podoplanin-CLEC-2 interaction is uncertain. In our studies, crosslinking of podoplanin on LEC (where there would be no substances from platelets) was similarly effective to platelets themselves, ruling out an absolute requirement for released agents in effects described. Others noted that knockdown of podoplanin in LEC reduced their ability to migrate across a wound or form networks on Matrigel [17,18]. In MDCK cells, overexpression of podoplanin increased active RhoA and phosphorylated ERM proteins [15], while in LEC, podoplanin knockdown was associated with a reduction in active RhoA but had no effect on the amount of phosphorylated ERM proteins [16] We did not find any change in phosphorylated ERM proteins after podoplanin knockdown, while crosslinking podoplanin did not inhibit the increase in active RhoA detected within 10 minutes or 10 hours after treatment with VEGFC (data not shown). Others have suggested that the action of podoplanin on ERM-phosphorylation operates via active RhoA [15], which would be consistent with the action of RhoA requiring podoplanin. Thus, two possibilities arise, in that podoplanin might be required for the activation of RhoA itself or may regulate RhoA function downstream of VEGF-R.

The observation that both loss of expression of podoplanin and crosslinking of endogenous podoplanin had similar effects on VEGF-induced migration suggests a requirement for signaling through podoplanin downstream of VEGF-R that would be turned off by podoplanin ligation. Interestingly, podoplanin crosslinking or knockdown solely abrogated VEGF-mediated migration, whereas responses to other growth factors remained unaffected. This could be explained if VEGFR signaling to RhoA required input from podoplanin, but signaling from the other growth factors to RhoA was via another route. It is also interesting that podoplanin is required in LEC to respond to VEGF-A or VEGF-C, while VEC which do not express podoplanin respond to VEGF-A at least. This difference may arise from the different combinations of VEGFR used by these cells (e.g., VEGFR1 and VEGFR2 in VEC vs. VEGFR2 and VEGFR3 in LEC) or from intrinsic differences in pathways downstream of the receptors in the different cells [42].

It is notable that we could not achieve better than ~50% reduction in surface expression of podoplanin using various combinations and concentrations of siRNA. However, this result is consistent with the level in other reports judging by the unquantified Western blots presented [17,18]. Nevertheless, this reduction in expression was sufficient to negate the effect of VEGF on migration. That 50% of podoplanin remained after 72 hour treatment with siRNA implies that there is a long-lived portion of the constitutive podoplanin. A possible explanation for the migration results is that there are “fast” and “slow” turnover pools of podoplanin that have different functional effects, or that the function of podoplanin changes with time after expression. Thus, the long-lived pool of podoplanin may not be linked to the signaling machinery for migration. However, the more newly expressed pool or fast-turnover pool (not expressed after treatment with siRNA) may be a key for the regulation of the VEGF response.

It is also notable that the network formation assay used here, based on the co-culture of LEC and HDF, is different from LEC networks formed on Matrigel. In the Matrigel assay, networks form on a surface of deposited growth factors and the networks typically only persist for a few hours [41]. In contrast, networks formed in co-cultures developed and persisted over four days. There is also evidence that for VEC at least, the networks have lumens, making this model more representative of endothelial tube formation *in vivo* [36]. These stable networks were clearly susceptible to the effects of platelets and podoplanin crosslinking. The transfilter assay used for the majority of the studies showed quite high levels of basal cell migration, which were relatively insensitive to treatments other than VEGF. However, the assay was robust in that we consistently observed significant effects of VEGF and modifiers of its response. Overall, in 23 experiments, the proportional increase from baseline with VEGF-C was 51 ± 8% (mean ± SEM). The finding that the basal migration was itself consistently unaffected by platelets or podoplanin crosslinking was a key in being able to show that these specifically affected the response to VEGF-C.

A number of observations indicate the physiological significance of the interactions analyzed here. Lack of podoplanin or platelet-specific loss of CLEC-2 causes malformation of the lymphatics during development in utero, where platelet-LEC interactions can be seen in the cardinal vein during the process of the separation of the lymphatic system [5,7]. In adult mice, radiation chimaeras reconstituted with CLEC-2-deficient bone marrow exhibit blood-lymphatic mixing in the intestines, illustrating a role for platelets in continuing repair and growth of the lymphatic system [12]. Interactions between platelets and LEC can also be observed near the junction of the thoracic duct and subclavian vein in mice, where they appear to prevent leakage of blood into the lymph [43]. Here, LEC migration was reduced when 10^7^ platelets (equivalent to the content of about 50µl of blood) were added to 3x10^4^ endothelial cells coating a surface area of 30mm^2^. In the assay, not all the platelets are likely to have actually adhered and made effective contact, while in vivo, there is likely to be a degree of flow, so that platelets are continually delivered, making many contacts possible. In flowing blood, we have shown that platelets are effectively marginated to the periphery close to endothelium [44] and that high levels of deposition of platelets occur when blood is flowed over LEC at low shear rates [45]. Thus, we believe that the conditions applied, although rather remote from those in vivo, are a relevant representation of interactions that could happen there, and that the changes in motility noted are relevant to the development, growth, and repair of lymphatics, which will include a component of LEC migration.

In conclusion, podoplanin which is highly expressed in LEC is an essential regulator of the response to VEGF in these cells, but not intrinsic motility or that stimulated by other growth factors. Platelets are thus able to modulate the migration of lymphatic endothelial cells and their ability to maintain stable networks by crosslinking this ligand which in turn modulates signaling through the RhoA pathway downstream of VEGFR-2 or -3. Our results suggest that podoplanin ligation can represent a novel approach to modulate LEC migration and tube formation in response to VEGF isoforms, key processes during cancer- and inflammation-induced lymphangiogenesis [42,46]. Further studies are warranted to dissect the potential therapeutic value of such an approach.
